# Dynamics of Natural Killer Cells Cytotoxicity in Microwell Arrays with Connecting Channels

**DOI:** 10.3389/fimmu.2017.00998

**Published:** 2017-08-17

**Authors:** Yuanhao Xu, Shufan Zhou, Yun Wah Lam, Stella W. Pang

**Affiliations:** ^1^Department of Electronic Engineering, City University of Hong Kong, Kowloon, Hong Kong; ^2^Center for Biosystems, Neuroscience, and Nanotechnology, City University of Hong Kong, Kowloon, Hong Kong; ^3^Department of Biology and Chemistry, City University of Hong Kong, Kowloon, Hong Kong

**Keywords:** natural killer cells, microwells, microchannels, cytotoxicity, cell interactions

## Abstract

Natural killer (NK) cells serve an important role in immune system by recognizing and killing the potentially malignant cells without antigen sensitization, and could be promising in cancer therapy. We have designed and fabricated microwell arrays with microchannel connections in polydimethylsiloxane (PDMS) substrates to study the interaction dynamics of NK-92MI cells with MCF7 breast cancer cells using time-lapse imaging by fluorescence microscopy for 15 h. Although cell seeding density was the same, NK cell cytotoxicity was found to be higher in larger microwells, which is manifested as increased target death ratio from 13.7 ± 3.1 to 46.3 ± 3.3% and shorter triggering time of first target lysis from 502 ± 49 to 391 ± 63 min in 150 μm × 150 μm microwells comparing to 50 μm × 50 μm wells in 15 h. Mirochannel connection between adjacent microwells of the same size increased the overall target death ratio by >10%, while connection between microwells of different sizes led to significantly increased target death ratio and delayed first target lysis in smaller microwells. Our findings reveal unique cell interaction dynamics, such as initiation and stimulation, of NK cell cytotoxicity in a confined microenvironment, which is different from population-based study, and the results could lead to a better understanding of the dynamics of NK cell cytotoxicity.

## Introduction

Natural killer (NK) cells are important immune cells in human body. Unlike other immune cells such as T-cells, these granular lymphocytes are identified by their ability to recognize and kill tumor or virally infected cells without antigen sensitization (1), which makes them very promising in anti-cancer therapy. NK cells can identify target cells by a variety of activating and inhibitory receptors at the NK cell immune synapse between NK cells and target cells (2). After target recognition, NK cells can kill target cells either by releasing chemokines to improve robust immune response (2), or by directly killing the target cell by lymphocyte-mediated lysing following a step of conjugation of NK cells to target cells, polarization of lytic granules toward target cells, and triggering of target cell degranulation (3, 4).

Natural killer cell activity can be meditated by a variety of chemokine (5). One of the most important chemokines to activate NK cell activities is interleukin-2 (IL-2), which is originally a T-cell growth factor for its capacity to maintain *in vitro* cultures of primary T cells (3). Clinical trials of NK cell-mediated tumor therapy have been reported by several medical research groups (6–9), and the results indicate that NK-92 cell line can be a good candidate for adoptive cellular immunotherapy since it does not have severe toxicities or side effect on tissues in patients (10, 11). However, NK cell-mediated therapy shows very diverse effect on different tumor tissues, which indicates that the NK cell activity can be tuned by its surrounding microenvironment. Thus, the activities of NK cells in different organs can be very complicated (12). To study the detailed effect of microenvironment on NK cell activity, *in vitro* study and imaging needed to be processed. However, NK cells co-cultured with target cells *in vitro* tend to accumulate and form a huge 3D spherical structure, which is extremely difficult to observe the detailed cell interactions in single cell level.

Natural killer cell migration and accumulation toward the infected tissue is also an important research area in NK cell immunotherapy. Movement of NK cell is known to be related to chemotaxis driven by a variety of chemokines, such as IFN-gamma and IL-2 (13–15). NK cell delivery to different organs by blood circulation is also known to be related to a complex mechanism, including IFN-gamma stimulation, T-cell regulation, and other chemokines in white mouse (16). Transwell migration assay is also widely applied to measure the mobility of NK cell *in vitro* (17, 18). However, due to the physical confinement by the extracellular matrix (ECM) in tissue, NK cells typically have limited opportunity to contact target cells. Thus, the actual NK cell–target cell interactions are processed in many separated “islands” rather than the commonly used 2-D *in vitro* culture platforms.

All these challenges require a solution to provide the control element needed to simplify NK cell–target cell interactions. In the past few years, platforms with microwell arrays for single cell analysis have been used to study NK cell heterogeneity in terms of cytotoxicity and migration behavior (19–23). Microengraving technique was also developed to monitor localized cell secretion (24, 25). However, reduced NK cell cytotoxicity in microwell assays were observed compared to population-based assays (26–28). This phenomenon may help to understand the varied NK cell effectiveness in clinical trials, but the mechanism is still not well studied. Here, microchips containing isolated and connected microwells with different sizes were developed to study the effect of confined microenvironment and cell number dependence on NK cell–target cell interactions. Most *in vitro* NK cell experiments used culture dishes with flat bottom. In the dishes, the NK cells were floating in the culture medium and roaming freely, which were very different from cells under *in vivo* conditions when they would be embedded in the ECM. Other NK cell studies in microwells focused on single cell interaction to reveal the heterogeneity of NK cells. In this study, by isolating cancer and NK cells into small groups and providing limited degree of connection through the channels in between the microwells, the platforms provided biomimetic microenvironment with controllable physical constraints for *in vitro* NK cell studies.

## Materials and Methods

### Microfabrication of Microwell and Microchannel Arrays on Polydimethylsiloxane (PDMS) Chips

**Figure 1:**
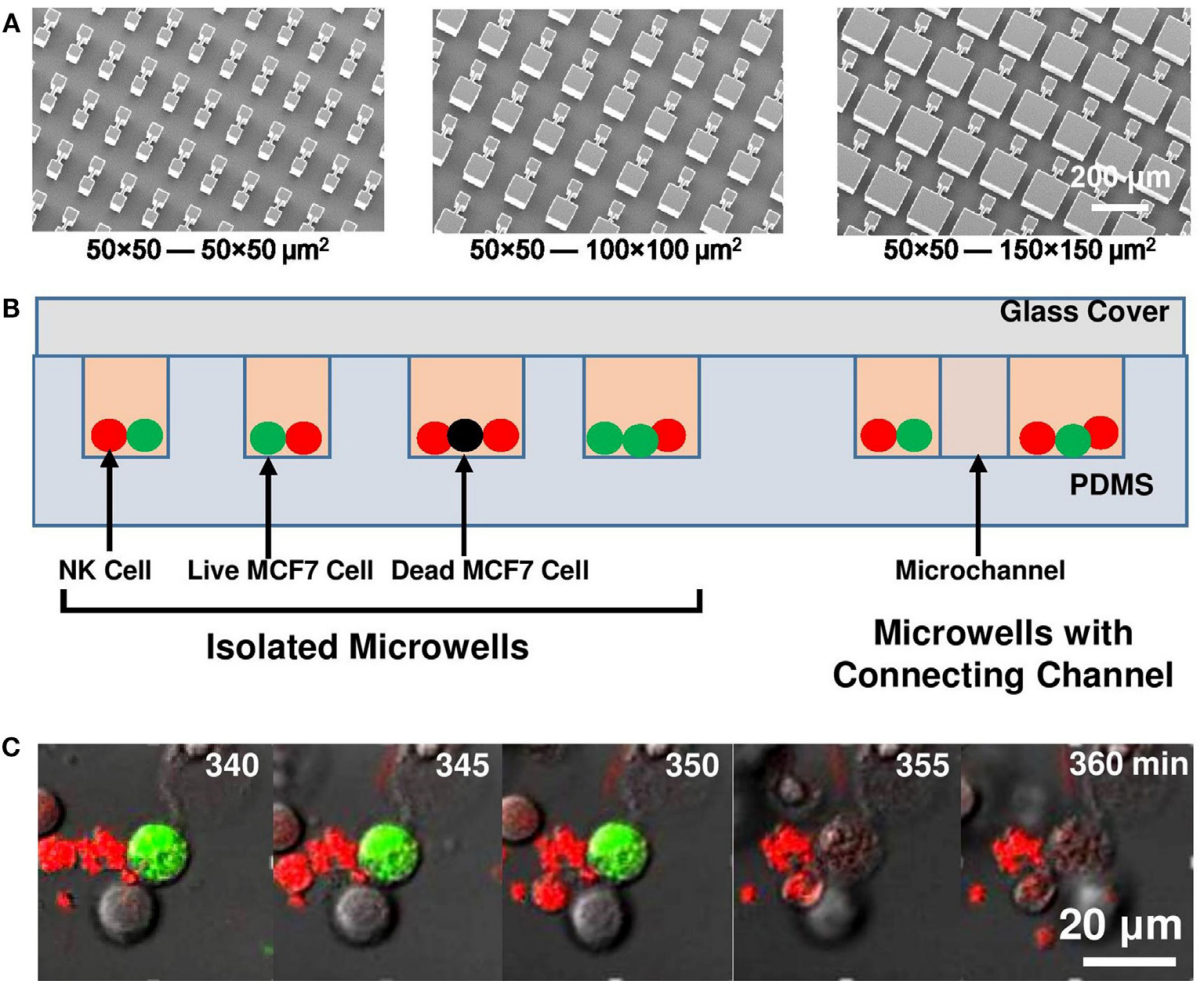
Experiment setup. **(A)** Micrographs of SU-8 stamps to duplicate polydimethylsiloxane (PDMS) platforms. **(B)** Schematic of microwell and microchannel arrays with natural killer (NK) cells (red) and MCF7 cells (green: live; black: dead) loaded. **(C)** Sequence of MCF7 cell lysis as MCF7 cell lost its green fluorescent protein signal at 355 min.

Microwells with 50 μm × 50 μm, 100 μm × 100 μm, and 150 μm × 150 μm sizes were designed to be either isolated or connected in pairs with 50 µm long, 10 µm wide microchannels. These microwells on PDMS chips were replicated from SU-8 mold on silicon wafer fabricated by UV patterning (350 nm wavelength) of 100 µm thick SU-8 2050 photoresist (MICROCHEM). The SU-8 molds (Figure 1A) were treated by 97% trichloro (1H,1H,2H,2H-perfluorooctyl) silane (FOTS) to form an anti-sticking layer and promote easy demolding of the PDMS replicas. An additional 10-µm-thick SU-8 layer was applied on silicon wafer to reduce thermal expansion stress between Si and SU-8 structures.

Polydimethylsiloxane replicas were fabricated by spin-coating PDMS pre-polymer (10:1 silicone elastomer base curing agent, Dow corning Sylgard 184) on the patterned Si molds. Curing of the PDMS pre-polymer was processed on a hotplate at 80°C for 4 h. Cured PDMS replicas were peeled off from the SU-8 molds and treated in a plasma using 20°sccm O_2_ at 80°mTorr and 50 W RF power for 3 min to form hydrophilic PDMS surface that helps culture medium and cells to be loaded into microwells more easily. The plasma-treated PDMS substrates were stored in deionized water immediately to maintain the hydrophilic property.

### Cell Culture

MCF7 breast cancer cells were maintained in Dulbecco’s modified eagle’s medium-high glucose (Invitrogen) with 10% [fetal bovine serum (FBS), Gibco], antibiotic-antimycotic (Gibco; 100 U/ml of penicillin, 100 µg/ml of streptomycin, and 0.25 µg/ml of amphotericin B), and supplemented with 2 mM alanyl-l-glutamine (Gibco) at 37°C and in 5% CO_2_ in a humidified incubator. MCF7 cells were transfected with a plasmid encoding green fluorescent protein using lipofectamine 2000 transfection reagent (Invitrogen) following the manufacturer’s instructions. The pcDNA3–EGFP plasmid was a gift from Doug Golenbock (Addgene plasmid # 13031).

Natural killer-92MI cells were purchased from American Type Culture Collection and maintained in a medium for human long-term culture of human cells (MyeloCult™ H5100, STEMCELL) with 2 mM l-glutamine and 1.5 g/l sodium bicarbonate. NK-92MI cells are maintained under 37°C with 95% air and 5% CO_2_, and passaged every other day to keep the cell concentration between 2 × 10^5^ and 1 × 10^6^ viable cells/ml in order to maintain the cell growth rate and NK cell cytotoxicity. NK cells were stained with calcein red-orange (CellTrace) following standard protocol before imaging to mark the cells. The condition of NK cells was constantly monitored to make sure that they were healthy before each experiment.

### Time-Lapse Imaging

Polydimethylsiloxane chips with different microwell designs were placed in 35 mm glass bottom confocal dishes (SPL) sterilized with 95% ethanol for 15 min and washed with phosphate buffered saline for four times before cell seeding. Microwells with various sizes were placed on the same dish so that they were imaged under the same experimental setting. MCF7 cells with total number of 5 × 10^5^ were seeded evenly into the dish and waited for 1 day to let them attach to the PDMS platform. The culture medium was then replaced to CO_2_ independent medium (Invitrogen 18045-088) with 10% FBS, antibiotic-antimycotic (Gibco; 100 U/ml of penicillin, 100 µg/ml of streptomycin, and 0.25 µg/ml of Amphotericin B), and supplemented with 2 mM alanyl-l-glutamine (Gibco). 7 × 10^5^ NK cells were then added into the dish and waited for 20 min for the cells to enter the microwells. Glass slip was placed on top of the PDMS chip to trap the cells inside microwells (Figure 1B). The seeded NK/target cell ratio in microwells was kept at 1.5–2. The whole setup was imaged with a laser scanning confocal microscope (Leica TCS SP5, with 488 nm Ar and 543 nm He–Ne visible laser) equipped with an incubation chamber at 37°C with humidified atmosphere. One image was collected every 5 min over a period of 15 h.

### Data Analysis

Time-lapse movies of four channels (green: 490–520 nm, red: 590–750 nm, bright field, and overlap channels) were exported and analyzed by NIH ImageJ (version 1.74v). Type and number of MCF7 and NK cells were identified and calculated from their color (green: MCF7 cells; red: NK cells) within first hour of time-lapse movie. Deaths of MCF7 cells were defined as permanent loss of fluorescence signal (Figure 1C). Number of MCF7 and NK cells, sequence and time of each MCF7 cell death case inside all individual microwells were recorded. MCF7 cell death ratio (*D*) was defined as number of MCF7 cell deaths (*N*_MCF7 Death_) divided by total number of MCF7 cells (*N*_MCF7 Total_); whereas NK cell killing efficiency (*K*) was defined as *N*_MCF7 Death_ per total number of NK cell (*N*_NK Total_). All the data were obtained based on over 10 repeated runs and the results were reproducible.

## Results

### NK and MCF7 Cell Interactions in Isolated Microwells

#### Decreased NK Cell Cytotoxicity Caused by Microwell Confinement

**Figure 2:**
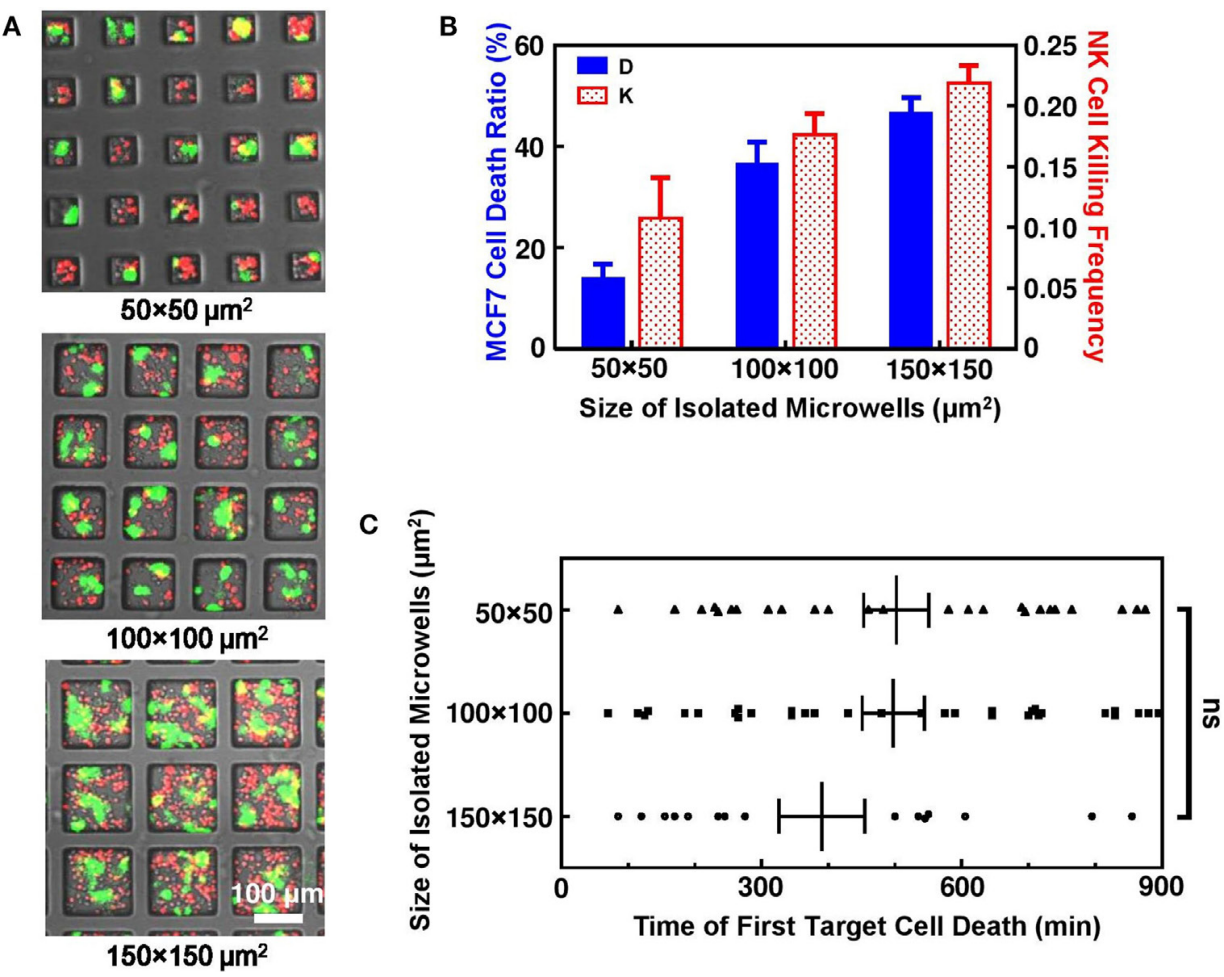
Interactions of natural killer (NK) and MCF7 cells in isolated microwells. **(A)** Microwells of different sizes with NK (red) and MCF7 (green) cells seeded at density of 1.2 × 10^5^ cells/cm^2^. **(B)** MCF7 cell death ratio and NK cell killing efficiency increased with microwell size. **(C)** Time for first MCF7 cell death was similar for all microwells. Scattered dots are individual cases of first MCF7 cell death and vertical lines are average values with error bars.

In this study, NK and target cells were seeded at the same ratio and density in the microwells. The actual NK/target cell ratio was not the same in microwells with different sizes because not all the cells were loaded into the microwells. However, an average NK/target cell ratio of 1.5–2 was obtained in all the experiments. Due to the randomness of cell loading in each individual microwell, cell number in different microwells varied, but generally there were 2–5 cells in 50 μm × 50 μm microwells, 10–18 cells in 100 μm × 100 μm microwells, and 25–40 cells in 150 μm × 150 μm microwells (Figure 2A). The overall NK cell cytotoxicity was similar in repeated experiments with the same microwell size and it varied with the size of the microwells even when the cells were seeded with similar cell density and NK/target cell ratio. These connected microwell designs were useful to study the effect of the surrounding microenvironment on NK cell activity.

Natural killer-92MI cells are known to be very effective NK cell line with multiple lytic granules that are capable for killing large amount of target cells within few hours of interactions inside culture dish with flat bottom. However, dramatically decreased NK cell cytotoxicity was observed inside isolated microwells. Overall, MCF7 cell death ratio was only 21.9% (*n* = 301) after 15 h cell interactions inside microwells. This is significantly lower compared to MCF7 death ratio with same NK and MCF7 cell density cultured on normal culture dish, where MCF7 cells can be completely killed by NK-92MI cells within 6 h under 2:1 effector to target mixing ratio. Such phenomenon of reduced NK cell cytotoxicity was also reported in other microwell-based NK cell study to achieve single NK cell target cell interaction pair (25). Comparison between microwells with different sizes showed that NK cell cytotoxicity increased with size of microwells where they were confined (Figure 2B). The increase of NK cell cytotoxicity was clearly illustrated by increased overall target cell death ratio and NK cell killing efficiency. However, there was no evidence that MCF7 cell deaths in larger microwells were triggered faster, indicating that the higher activity of NK cells in microwells of 100 μm × 100 μm and 150 μm × 150 μm sizes was caused by fast serial killing after triggering of first kill (Figure 2C).

#### NK Cell Cytotoxicity Increases with Total Number of Cells Confined in Single Microwell

Two hypotheses were made to explain the deficiency of NK cell activity with microwell confinement. First hypothesis assumed that NK cells need to find the correct spots to recognize surface receptors on target cells. With microwell confinement, MCF7 cells cannot fully spread themselves and large surface area of them was protected by sidewalls of microwells, decreasing the available spots for NK cells to attack. The other hypothesis highlighted the importance of enough target cells to stimulate NK cell activity. Microwells can split cells into small groups and avoid transition between adjacent microwells; thus, NK cell–MCF7 cell interactions can only happen within the small group confined together, reducing the chance of NK cells to be activated.

An extra experiment was carried out to verify the first hypothesis. This time, different cell seeding density was applied to microwells with different sizes. The final goal was to achieve similar cell number in different microwells with approximately 2:1 effector to target mixing ratio (Figure S1 in Supplementary Material). Experiment result showed similar NK cell cytotoxicity in these cases, suggesting that confined surface area of target cells was not the major factor that affects NK cell cytotoxicity.

**Figure 3:**
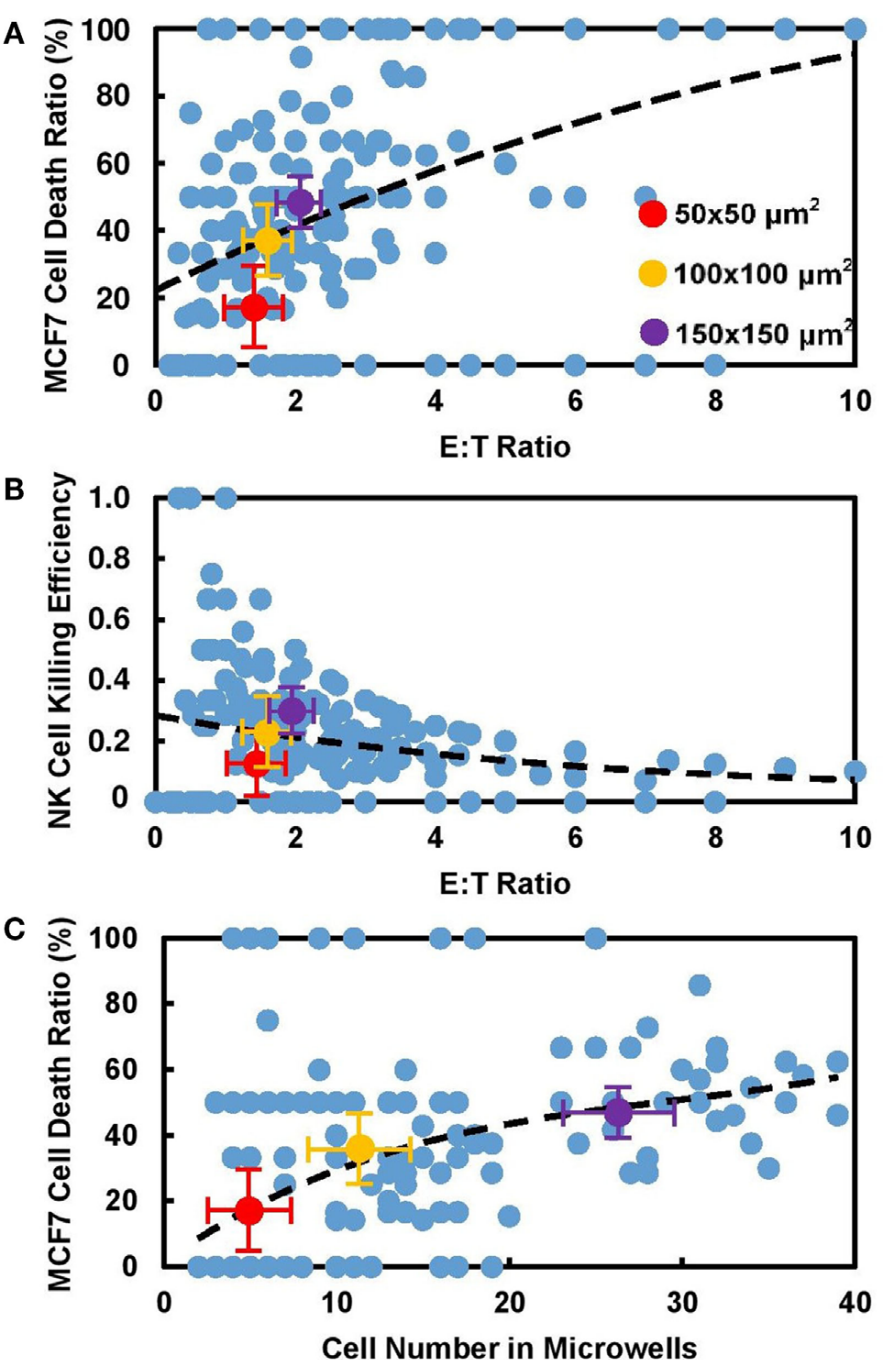
Cytotoxicity of natural killer (NK) cell increased with number of cells confined in microwells. Experiment data obtained from all individual microwells (blue) and average value from 50 μm × 50 μm (red), 100 μm × 100 μm (yellow), and 150 μm × 150 μm (violet) microwells were displayed. **(A)** MCF7 cell death ratio increased with E:T ratio in microwells. **(B)** Killing capability of NK cells decreases with E:T ratio in corresponding microwells. Larger dots show average values in different sizes. **(C)** MCF7 cell death ratio increased with number of cells in microwells regardless of their sizes.

Effector (E) to target (T) ratio is defined as ratio of NK cell number to MCF7 cell number, which is usually regarded as an important parameter that affects NK cell cytotoxicity in population-based study. While our correlation of E:T ratio with MCF7 cell death ratio (Figure 3A) and NK cell killing efficiency (Figure 3B) in each individual microwell shows similar trend with population-based study (28), it also shows that NK cell activity is different in microwells with various sizes even with similar E:T ratio. Total number of cells confined in individual microwells is found to be directly related to NK cell cytotoxicity in the corresponding microwell. Figure 3C shows the mapping of total cell number and NK cell death ratio in all individual microwells. Although large variation is observed, overall trend shows increased NK cells cytotoxicity with larger group of cells confined together. This trend remained the same when 0 and 100% MCF7 cell death cases were excluded (Figure S2 in Supplementary Material), which showed that the results were not biased by the limited target cell number in the small microwells. Previous research on “serial killer” NK-92MI cells revealed the importance of enough simulation signals from NK cell–target cell contacts to trigger the cytotoxicity of NK cells (28–31). Our result showed that first target lysis can happen very late (300–600 min) compared to some other population-based results (~70 min), which can be explained by deficiency in activation of NK cells due to lack of targets available.

### NK and MCF7 Cell Interactions in Connected Microwells

#### Connection between Microwells Synchronize NK Cells Cytotoxicity

**Figure 4:**
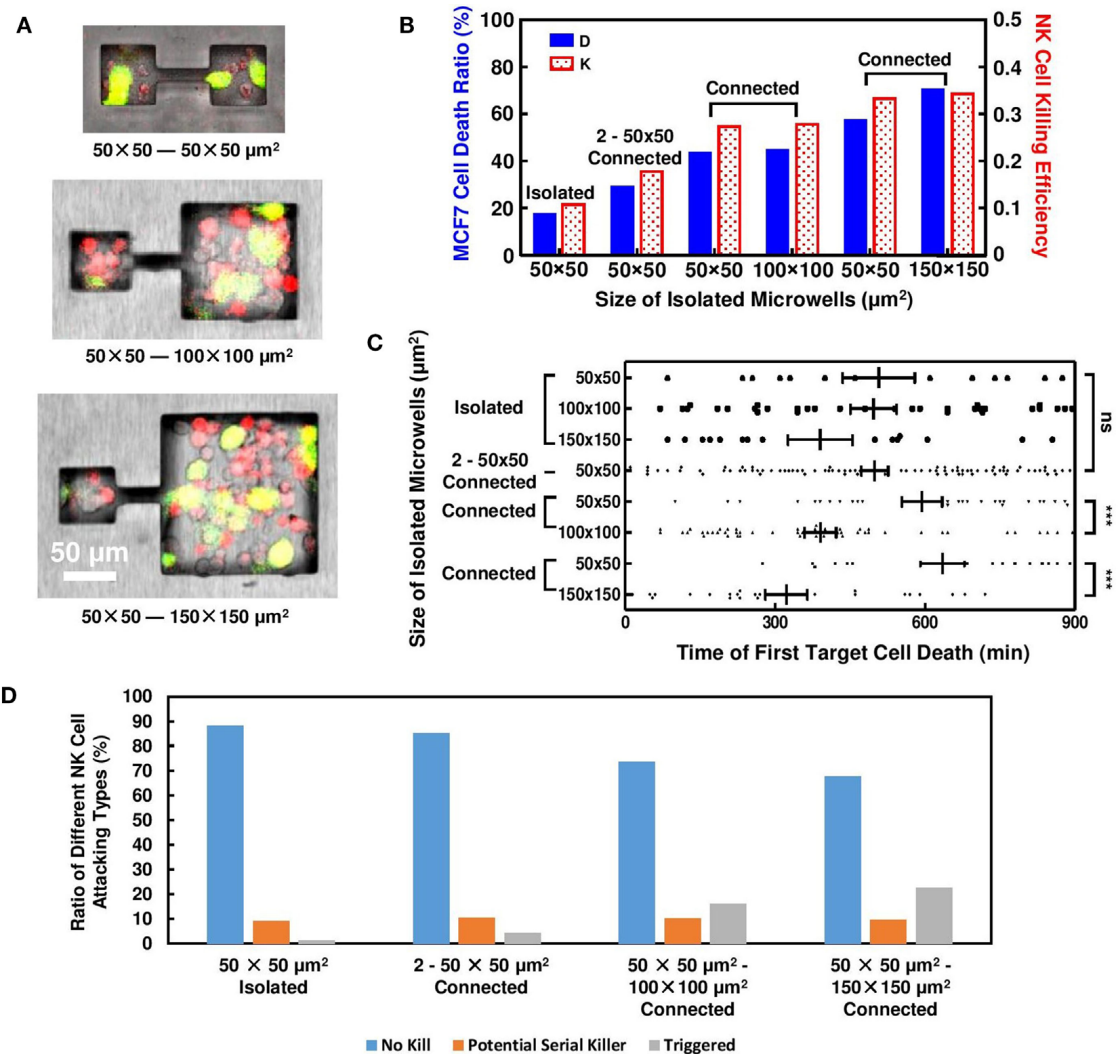
Natural killer (NK) cell cytotoxicity in connected microwells. Results obtained from isolated microwells are listed for comparison. **(A)** MCF7 and NK cells distributions in microwells connected by 50-µm long, 10-µm wide microchannels. **(B)** Connection between 50 μm × 50 μm microwells with microwells of larger sizes increased NK cell cytotoxicity. **(C)** Time for first MCF7 cell death in connected microwells; in 2–50 μm × 50 μm microwells, triggering time was similar to isolated wells; when connected to larger microwells, triggering time of first target cell death in 50 μm × 50 μm microwells was delayed. **(D)** Proportion of NK cell in different attacking modes for isolated and connected microwells. More NK cells in triggered mode were observed when 50 μm × 50 μm microwells were connected to 100 μm × 100 μm and 150 μm × 150 μm microwells.

Another set of experiments was established to connect microwell pairs with 10-µm wide, 50-µm long microchannels (Figure 4A). NK cell activities in these connected microwells were recorded and compared to their isolated counterparts. The microchannel created line of sight path between adjacent microwells and provided similar microenvironment in the connected microwells. Due to the limitation of channel width, MCF7 cells cannot migrate across the connected microwells, but a small population of the NK cells could squeeze through the channels easily and migrate to the adjacent microwells. The NK cells had unidirectional migration between the connected microwells with different sizes. 84.4% (*n* = 32) and 88.7% (*n* = 53) of the NK cells migrated from the smaller microwells toward the larger ones for the 50 μm × 50 μm–100 μm × 100 μm and 50 μm × 50 μm–150 μm × 150 μm connected pairs, respectively. Since the probability for NK cells to migrate from larger microwells to smaller ones was low, no target cell killing was observed for any NK cells migrating from the larger microwells to the 50 μm × 50 μm microwells. Meanwhile, the channel also provided diffusion path for multiple types of cell secretion.

Natural killer and MCF7 cells were seeded into the microwells with similar density as the previous experiment. Although the initial E:T ratio in the connected microwells remained an average value of 2:1 shortly after cell seeding, NK cells had a preference to migrate into the larger microwells, resulting in different steady-state E:T ratio. The increased E:T ratio in 100 μm × 100 μm and 150 μm × 150 μm microwells lead to enhanced NK cell cytotoxicity compared to results obtained from isolated cases. NK cell cytotoxicity in the connected 50 μm × 50 μm microwells also increased to similar level, which is far larger compared to isolated cases (Figure 4B). Microchannel connection also results in delayed first target lysis in 50 μm × 50 μm microwells (Figure 4C), where in isolated cases such phenomenon cannot be observed.

Late triggering of target lysis by NK cell is usually associated with NK cell deficiency (32, 33). However, our experiment utilizing connected microwells showed decreased E:T ratio, delayed first target lysis, yet increased NK cell cytotoxicity in 50 μm × 50 μm microwells. The result indicated that NK cell activity in 50 μm × 50 μm microwells must be stimulated during this process, and the stimulation signal came from the adjacent microwells connected to them. NK cell–target cell contacts in 50 μm × 50 μm microwells were monitored and compared (Figure S3 in Supplementary Material). End of an NK cell contact with the target was defined when the NK cell switched to attack another target cell. In isolated microwells, most NK cells failed to kill any MCF7 cells during the 15 h tracking period using the confocal microscope. 10% of the NK cells were potential “serial killer” cells that had killed all target cells they have contacted or had killed their first target and continued to attack the next one. When the 50 μm × 50 μm microwells were connected to other wells, while some NK cells maintained their serial killing capability, some previously inactive NK cells began to lyse target cells after a few failed attempts (Figure 4D). The proportion of NK cells with such “triggering” behavior increased with the connected microwell size. The NK cells in the “triggering” mode could be related to the delayed first target cell deaths and the increased target cell death ratio when the 50 μm × 50 μm microwells were connected to other microwells. The proportion of potential serial killer NK cells was around 10% for all microwell configurations as shown in Figure 4D. Due to the limited number of target cells in the small microwells, some of these NK cells had not shown their full potential to kill multiple target cells yet. In the 100 μm × 100 μm and 150 μm × 150 μm microwells, these potential “serial killers” could kill additional target cells which resulted in the larger overall target cell death ratio.

#### Each Target Death Increase NK Cell Cytotoxicity in Surrounding Microenvironment

**Figure 5:**
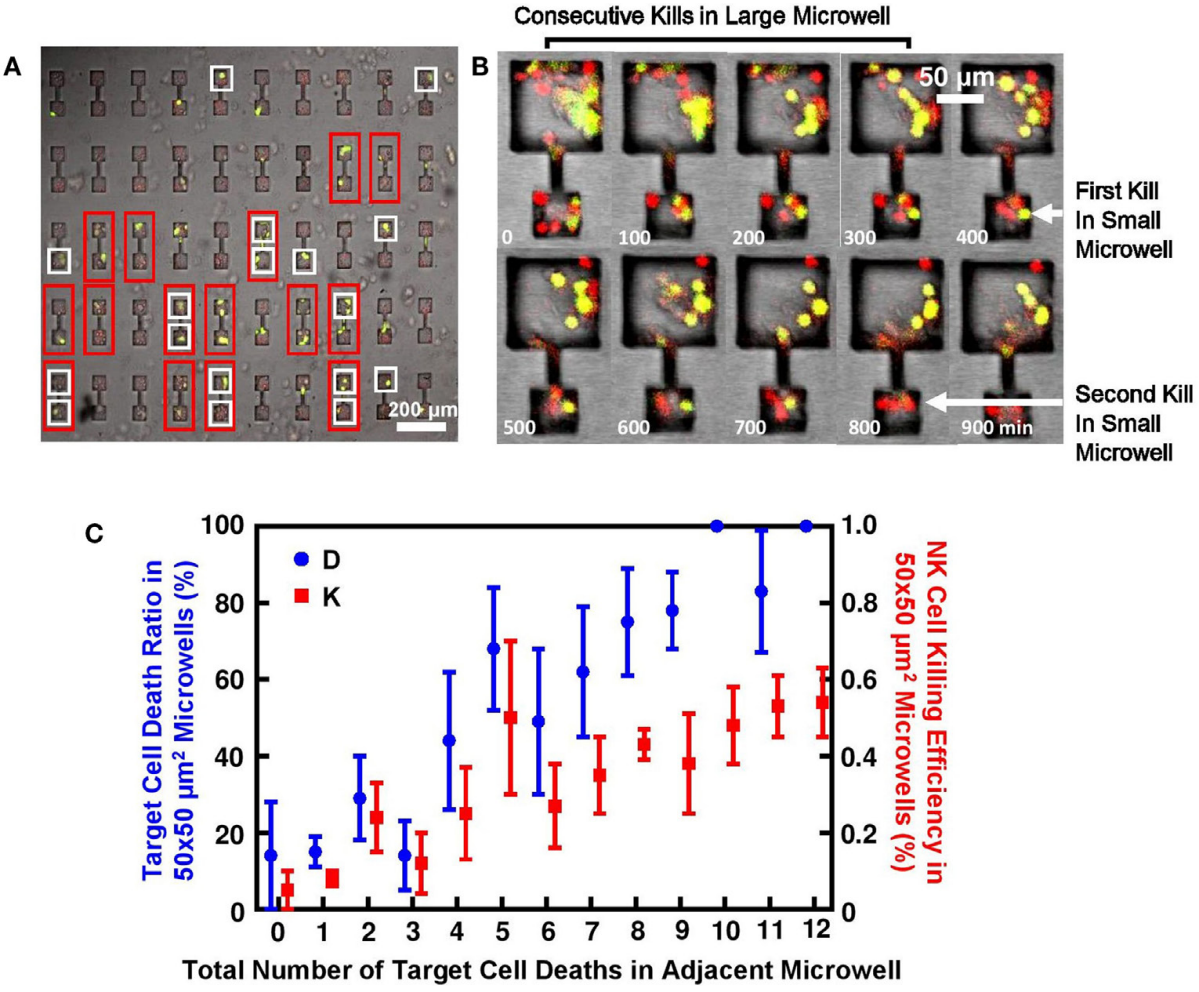
Target lysis by natural killer (NK) cells directly affected cell deaths in adjacent microwell connected by microchannel. **(A)** MCF7 cells had high tendency to die in pairs in 2–50 μm × 50 μm connected microwells. Microwell pairs outlined by red frame had MCF7 cells successfully seeded in both sides; microwells outlined by white frame had at least one target lysis; no target death is observed in remaining microwells. **(B)** Time sequence of MCF7 cells being attacked in connected microwells. First kill in 50 μm × 50 μm microwell happened after consecutive target cell lysis in 100 μm × 100 μm microwell. **(C)** Target cell death in microwell increased NK cell cytotoxicity in connected microwell. *Y*-axis is MCF7 cell death ratio *D* and NK cell killing efficiency E in 50 μm × 50 μm microwells; *X*-axis is number of target lysis in adjacent larger microwell.

Increased NK cell cytotoxicity in 2–50 μm × 50 μm connected microwells can be explained by “paired” target cell deaths. Overall, MCF7 cell death ratio is still relatively low in this design (26%), and MCF7 cell deaths can only be observed in specific microwells. However, these deaths were very likely to happen in pairs (Figure 5A). When target cell lysis happened in one microwell, the target cells in adjacent microwell would also die afterward. These paired deaths increased overall target death ratio and NK cell killing efficiency.

In 50 μm × 50 μm microwells connected to larger microwells with larger sizes, similar explanation can also apply. Figure 5B shows typical target cell death sequence in the connected microwells. First death in 50 μm × 50 μm microwells will happen after a series of deaths in the larger microwells, which also explains the significantly different triggering time of first target lysis (Figure 4C). Statistics show that NK cell cytotoxicity in 50 μm × 50 μm microwells is affected by total number of target cell deaths in the adjacent microwells (Figure 5C). Although large variation is observed, increased number of MCF7 cell deaths in larger microwells can generally lead to higher NK cell cytotoxicity in the smaller microwells represented both in MCF7 cell death ratio and NK cell killing efficiency.

All these results showed clear evidence that NK cell cytotoxicity can be enhanced by the deaths of adjacent target cells, the key problem is how they are stimulated. The fact that this simulation signal can be easily manipulated by microchannel connection suggested that diffusion of cytokines and biomolecules was the most likely method of signal transmission. Increased NK cell cytotoxicity could be a result of either enhanced NK cell activity or decreased target cell susceptibility. Various studies in the past showed the importance of chemokines, such as IL-2 (5, 34, 35) and IFN-gamma (36). But the type of NK cells (NK-92MI) we used in this study is well known to be genetically modified and capable of secreting IL-2 themselves (37); thus, IL-2 does not seem to be the major factor. However, the fact that cytotoxicity of NK-92MI cells was much lower in 50 μm × 50 μm microwell confinement may suggest that the IL-2 secretion of individual NK-92MI cell could have large variations. When NK cells are in large numbers, NK cell activity can be enhanced by IL-2 secreted from neighboring cells. But when NK cells are isolated individually, NK cell may be less active due to limited IL-2 presence.

Studies of “serial killer” NK cells stated the importance of enough NK cell–target cell contacts for NK cells to recognize the target cells and trigger fast lysis. But the very fact that serial killing required long triggering time of first kill followed by rapid consecutive kills may also indicate the importance of target cell death in activation of NK cell activity. Bystander effect is a well-known topic in cancer biology. Recent studies have shown that release of several components inside cell membrane after apoptosis can lead to reduced cell susceptibility to stress in microenvironment (38–40). Such effect can explain the correlation of number of target cell death versus MCF7 cell death ratio in 150 μm × 150 μm–50 μm × 50 μm and 100 μm × 100 μm–50 μm × 50 μm connected microwells.

## Discussion

Natural killer-92MI cells have been commonly regarded as one of the most active type of NK cells, which are capable of killing target cells rapidly within few hours in *in vitro* experiments. With our various designs of microwells and microchannels, we showed the dynamics of NK cell activity within this cell line, and how their cytotoxicity can be adjusted by different level of microwell confinement and microchannel connections. Different sizes of microwells trapped different number of cells inside. In smaller microwells, NK cell cytotoxicity was significantly reduced due to their incapability of finding the “correct” target cell to kill; with increased microwell size, more complex interactions increase the probability of NK cells to form effective interaction pairs with target cells, resulting in promoted NK cell activity. Target cell death can also provide conditioned medium that decrease the viability of other target cells in the surrounding microenvironment through the bystander effect, resulting in significantly enhanced overall NK cell cytotoxicity in this region. Microchannel connections provided path for diffusion of cytokines and biomolecules and enhanced overall NK cell cytotoxicity. NK cell cytotoxicity in platforms with microchannel connections was much higher compared to NK cells confined in isolated microwells. These cells with different degrees of confinement showed much lower cytotoxicity than NK cells in typical culture dishes.

Deficiency of NK cells in clinical trials compared to *in vitro* experimental results has been a major roadblock in NK cell-related cancer therapy. While most of the recent NK cell studies focus on population-based or single cell interactions, cell interactions in small groups may be the missing link. Since cancer cells in tumor are separated into small groups by ECM, microwells with different sizes and connections can provide a better biomimetic platform for cancer immunology study. On the other side, high NK and target cell seeding densities were required in order to achieve homogeneous cell seeding in the microwells with different sizes. The NK cell–target cell interactions were complex and could have frequent z-direction movement, which made it difficult to identify all NK cell–target cell conjugations. The cells were also in contact or in close proximity with each other, which made it difficult to separate bystander killing from direct NK cell killing. In this case, extensive manual tracking of the cell behaviors was needed in this study due to the complex cell interactions that made it difficult to apply computer-assisted tools for cell viability analysis. The NK-92MI cell line utilized in this study was specifically modified for fast and effective cancer cell treatment, whereas further studies using normal NK cells isolated from peripheral blood in these connected microwells will be also be important. However, these analyses were effective in evaluating the effects of physical constraints on NK cell–target cell interactions and the dynamics of NK cell cytotoxicity in a systematical way. Thus, the platforms presented here could provide insight for developing effective immunotherapy methods in the future.

## Author Contributions

YX conducted a major part of the experiments, analyzed data, designed and fabricated the microchips, and wrote the article. SZ performed some of the experiments, analyzed data, and fabricated microchips. YL provided biological and imaging support. SP conceptualized experimental principle, designed the study, and wrote the paper.

## Conflict of Interest Statement

The authors declare that the research was conducted in the absence of any commercial or financial relationships that could be a potential conflict of interest.
