# Supplementary material for: Dynamics of Natural Killer Cells Cytotoxicity in Microwell Arrays with Connecting Channels
Source: Front Immunol. 2017 Aug 17;8:998. doi: 10.3389/fimmu.2017.00998 (PMC5562680; doi:10.3389/fimmu.2017.00998)
Supplement: Supplementary file 1 [file image_1.pdf]

*Supplementary Material*

# Dynamics of natural killer cells cytotoxicity in microwell arrays with connecting channels

**Yuanhao Xu[1,2], Shufan Zhou[1,2], Yun Wah Lam[2,3], and Stella W. Pang[1,2*]**

[1]Department of Electronic Engineering

[2]Center for Biosystems, Neuroscience, and Nanotechnology

[3]Department of Biology and Chemistry

City University of Hong Kong, Hong Kong

***Correspondence:**
S. W. Pang
pang@cityu.edu.hk

## 1 Supplementary Figures

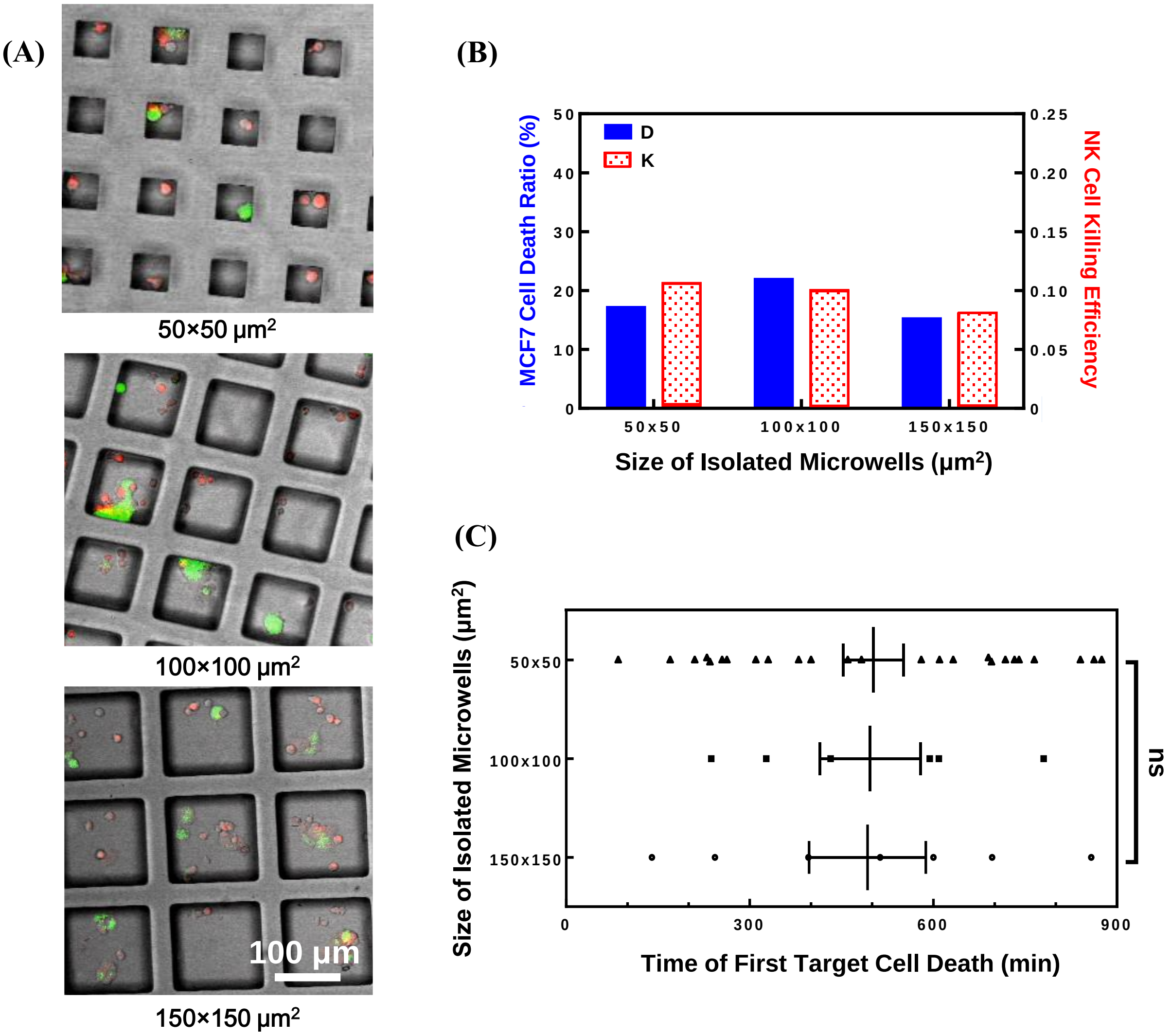


**Supplementary Figure 1. Interactions of NK and MCF7 cells in isolated microwells with similar cell seeding number. (A)** Microwells with different sizes trapping similar number of cells with $1.3\times10^4$ cells/cm$^2$ seeding density. **(B)** MCF7 cell death ratio and NK cell killing efficiency were similar. **(C)** Triggering time of first MCF7 cell death was similar for all microwells. Scattered dots are individual cases of first MCF7 cell death and vertical lines are average values with error bars.

(A)

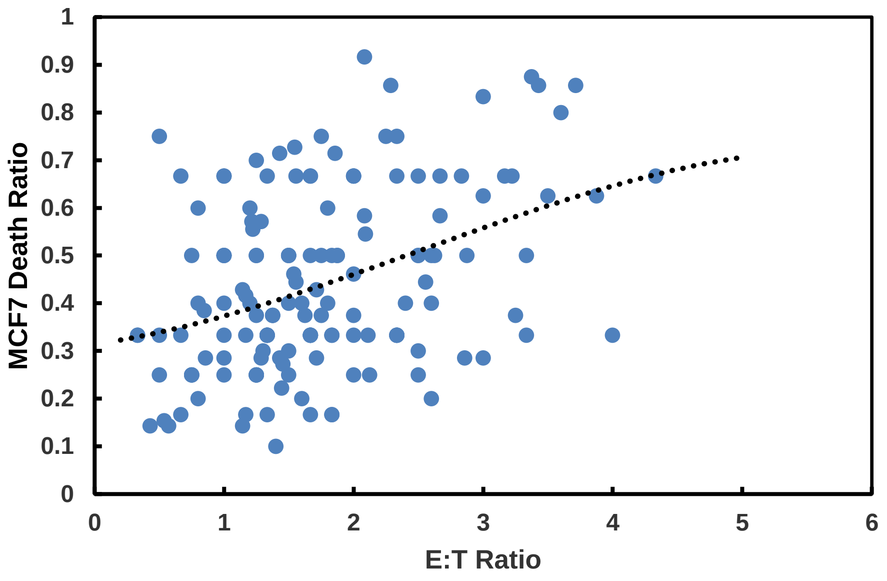


(B)

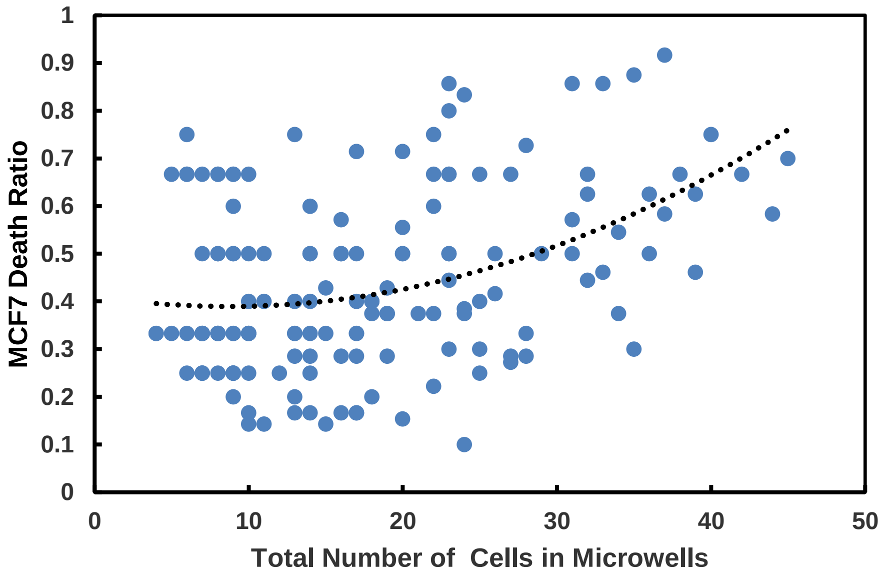


**Supplementary Figure 2. Cytotoxicity of NK cell increased with number of cells confined in microwells with 0% and 100% data points excluded.** Experimental data obtained from all microwells except those with 0% or 100% death ratio were displayed. **(A)** MCF7 cell death ratio increased with E:T ratio in microwells. **(B)** MCF7 cell death ratio increased with number of cells in microwells regardless of their sizes.

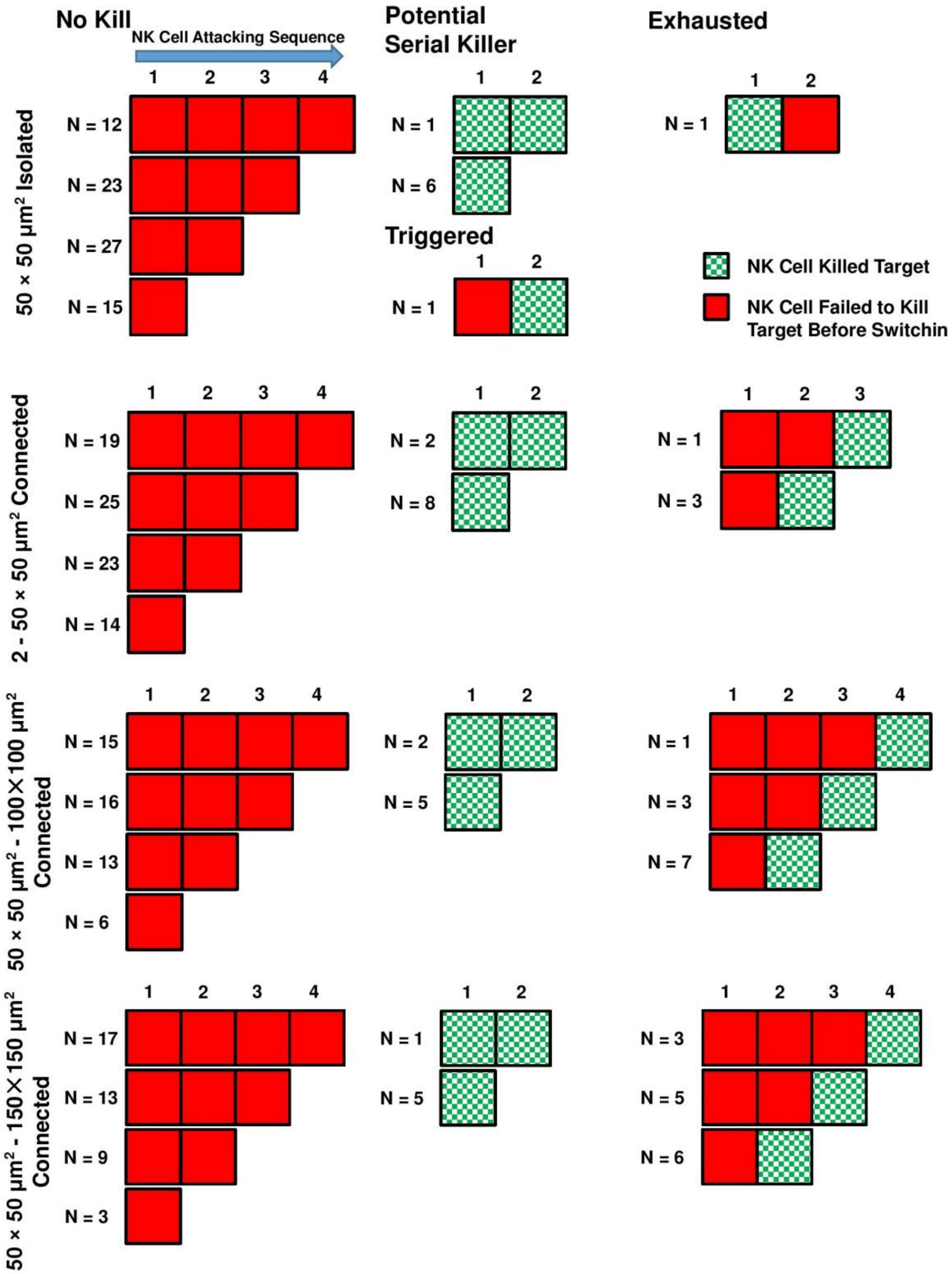


**Supplementary Figure 3. NK cell attacking sequence patterns in different microwell configurations.** Each NK cell attack was defined as NK cell attacking one target cell. NK cell switching to attack another target cell was defined as start of new attack. NK cell attacking sequence patterns were categorized into different modes based on whether NK cell successfully killed target cell upon each contact. NK cell that killed target cell it contacted was defined as potential "serial killer" cell since it could subsequently kill more target cells.
